# Artificial intelligence–based skeletal muscle estimates and outcomes of EUS-guided treatment of pancreatic fluid collections

**DOI:** 10.1016/j.igie.2024.06.006

**Published:** 2024-07-01

**Authors:** Mamoru Takenaka, Wataru Gonoi, Tatsuya Sato, Tomotaka Saito, Shouhei Hanaoka, Tsuyoshi Hamada, Shunsuke Omoto, Atsuhiro Masuda, Masahiro Tsujimae, Takuji Iwashita, Shinya Uemura, Shogo Ota, Hideyuki Shiomi, Toshio Fujisawa, Sho Takahashi, Saburo Matsubara, Kentaro Suda, Akinori Maruta, Kensaku Yoshida, Keisuke Iwata, Mitsuru Okuno, Nobuhiko Hayashi, Tsuyoshi Mukai, Hiroyuki Isayama, Ichiro Yasuda, Yousuke Nakai, WONDERFUL study group in Japan and collaborators, Arata Sakai, Ryota Nakano, Yuhei Iwasa

**Affiliations:** 15 Division of Gastroenterology, Department of Internal Medicine, Kobe University Graduate School of Medicine, Hyogo, Japan; 16 Division of Hepatobiliary and Pancreatic Diseases, Department of Gastroenterology, Hyogo Medical University, Hyogo, Japan; 17 Department of Gastroenterology, Gifu Municipal Hospital, Gifu, Japan; 1 Department of Gastroenterology and Hepatology, Faculty of Medicine, Kindai University, Osaka, Japan; 2 Department of Radiology, Graduate School of Medicine, The University of Tokyo, Tokyo, Japan; 3 Department of Gastroenterology, Graduate School of Medicine, The University of Tokyo, Tokyo, Japan; 4 Department of Hepato-Biliary-Pancreatic Medicine, Cancer Institute Hospital, Japanese Foundation for Cancer Research, Tokyo, Japan; 5 Division of Gastroenterology, Department of Internal Medicine, Kobe University Graduate School of Medicine, Hyogo, Japan; 6 First Department of Internal Medicine, Gifu University Hospital, Gifu, Japan; 7 Division of Hepatobiliary and Pancreatic Diseases, Department of Gastroenterology, Hyogo Medical University, Hyogo, Japan; 8 Department of Gastroenterology, Graduate School of Medicine, Juntendo University, Tokyo, Japan; 9 Department of Gastroenterology and Hepatology, Saitama Medical Center, Saitama Medical University, Saitama, Japan; 10 Department of Gastroenterology, Gifu Prefectural General Medical Center, Gifu, Japan; 11 Department of Gastroenterology, Gifu Municipal Hospital, Gifu, Japan; 12 Third Department of Internal Medicine, University of Toyama, Toyama, Japan; 13 Department of Gastroenterological Endoscopy, Kanazawa Medical University, Ishikawa, Japan; 14 Department of Endoscopy and Endoscopic Surgery, The University of Tokyo Hospital, Tokyo, Japan

## Abstract

**Background and Aims:**

Skeletal muscle status may affect clinical outcomes of a variety of pancreatic diseases. Skeletal muscle quality and quantity have not been fully examined in relation to the outcomes of EUS-guided treatment of pancreatic fluid collections (PFCs).

**Methods:**

Using a multi-institutional cohort of 372 patients receiving EUS-guided treatment of PFCs in 2010 to 2020, we examined the association of skeletal muscle status with adverse outcomes, including clinical treatment failure and in-hospital mortality. We used an in-house deep learning–based platform for preprocedural CT images, and skeletal muscle density (SMD) and skeletal muscle index (SMI; height-adjusted muscle area) were calculated as surrogates for muscular quality and quantity, respectively. Multivariable logistic regression analysis was conducted to calculate odds ratios (ORs) for adverse outcomes.

**Results:**

Lower-level SMD was associated with higher risks of clinical failure and in-hospital mortality (*P*_trend_ < .001). The adjusted OR for clinical failure comparing the extreme quartiles was 3.64 (95% confidence interval, 1.52-8.72). Compared with patients in the top 2 quartiles, patients in the lowest quartile had an adjusted OR for in-hospital mortality of 12.4 (95% confidence interval, 3.43-44.8). No effect modification according to the PFC types on the SMD–outcome relationship (*P*_interaction_ > .16) was observed. SMD was not associated with the risk of procedure-related adverse events or PFC recurrence. SMI was not associated with adverse outcomes (*P*_trend_ > .39).

**Conclusions:**

In patients with endoscopically managed PFCs, SMD (but not SMI) was associated with the risks of clinical failure and in-hospital mortality, supporting the prognostic role of skeletal muscle quality.

Pancreatic fluid collections (PFCs) primarily encompass walled-off necrosis (WON) and pancreatic pseudocysts, which develop as local adverse events of acute pancreatitis.^1^, ^2^, ^3^ When conservative management fails to alleviate PFC-related symptoms, patients are typically directed toward drainage-based interventions. The growing popularity of EUS-guided transluminal interventions positions endoscopic procedures as a first choice for managing symptomatic PFCs in many centers.^4^, ^5^, ^6^ The evolving approach of using a lumen-apposing metal stent (LAMS) has transformed nonsurgical management by providing a transluminal port for endoscopic necrosectomy following EUS-guided drainage of WON.^7^, ^8^, ^9^, ^10^, ^11^

Despite the increasing safety and effectiveness of EUS-guided treatment, a noteworthy number of patients experience procedure-related adverse events and fatal outcomes.^12^, ^13^, ^14^ The invasive nature of the procedure and the compromised health of the treated patients underscores the importance of identifying predictive factors for the unfavorable clinical outcomes of EUS-guided PFC drainage and adjunctive interventions. To date, risk stratification has primarily relied on information on the characteristics of PFCs (eg, necrosis proportion, PFC extension status) and procedures (eg, necrosectomy).^14^, ^15^, ^16^ A better understanding of patient characteristics linked to unfavorable outcomes of EUS-guided PFC treatment would aid in clinical decision-making and the refinement of the treatment protocol for patients undergoing this therapeutic approach.

Sarcopenia is an aging-related or secondary loss of skeletal muscle volume and functionality that has been implicated in worse clinical outcomes of various acute and chronic conditions.^17^^,^^18^ In the field of pancreatology, clinical studies suggest that impaired skeletal muscle status may be correlated with a risk of in-hospital mortality among patients with acute pancreatitis and prognosis among patients with pancreatic cancer.^19^, ^20^, ^21^ Sarcopenia might serve as a surrogate for various factors with negative impacts on survival times, including impaired nutritional status, unfavorable metabolic alterations, and sustained systemic inflammation.^22^, ^23^, ^24^, ^25^ Conversely, decreased levels of muscular quality and quantity may cause sustained inflammatory reactions and dysregulate the systemic immune response.^24^^,^^25^ The appropriate management of patients’ general and immune conditions plays a pivotal role in the clinical success of EUS-guided treatment of PFCs.^26^ We therefore hypothesized that skeletal muscle quality and quantity might be associated with the risks of adverse outcomes in patients receiving EUS-guided treatment of PFCs.

To test our hypothesis, we used a multi-institutional cohort of patients receiving EUS-guided PFC treatment, which was established within the WONDERFUL (WON and Peripancreatic Fluid Collection) consortium in Japan.^27^^,^^28^ Skeletal muscle density (SMD) and area at the time of the initial EUS-guided drainage (as surrogates for their quality and quantity, respectively) were examined in relation to adverse outcomes. To overcome the potential biases due to manual annotation of skeletal muscle areas on cross-sectional imaging studies, an artificial intelligence (AI)-driven platform was used for the automatic annotation of skeletal muscle areas on preprocedural CT images.^29^

## Methods

### Study population

**Figure 1:**
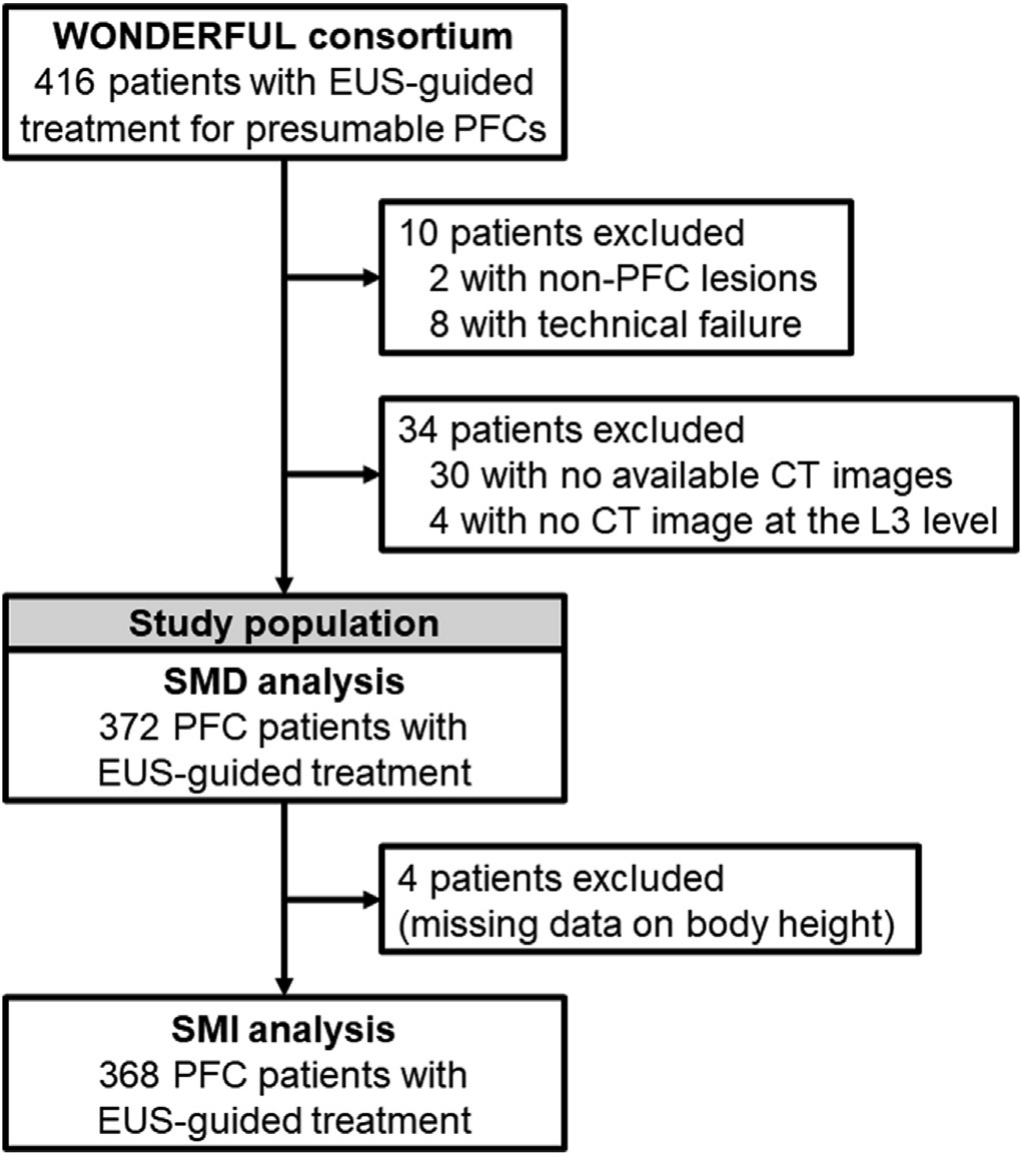
Flow diagram showing selection of patients receiving EUS-guided treatment of pancreatic fluid collections (PFCs) in a multi-institutional cohort within the WONDERFUL (WON and Peripancreatic Fluid Collection) consortium. *L3*, Third lumbar vertebra; *SMD*, skeletal muscle density; *SMI*, skeletal muscle index.

Within the WONDERFUL cohort derived from 10 high-volume centers in Japan (Appendix 1, available online at www.igiejournal.org), data were collected on consecutive patients who received EUS-guided treatment of PFCs from January 1, 2010, through November 30, 2020 (Fig. 1). PFCs were categorized as having WON or pancreatic pseudocysts according to the revised Atlanta classification.^3^ Patients with technical failure in EUS-guided drainage and patients with non-PFC lesions managed via EUS-guided drainage were excluded.

Using a standardized study database constructed via the Microsoft Access software (Microsoft Japan, Tokyo, Japan), study physicians reviewed medical charts and collected the following clinical data: patient demographic characteristics, radiologic features of PFCs, details of all endoscopic and nonendoscopic procedures, treatment outcomes (including adverse events), and PFC recurrence during follow-up. The patients were followed up until death or the end of follow-up (December 31, 2023), whichever came first.

The current study was conducted according to the guidelines in the Declaration of Helsinki. Written informed consent for the procedure was obtained from all patients, and consent for the use of the retrospective data for research was obtained on an opt-out basis. The study was approved by the ethics committee at each center and was registered with UMIN-CTR (registration number, UMIN000044130).

### Deep learning–based assessment of skeletal muscle status using preprocedural CT images

**Figure 2:**
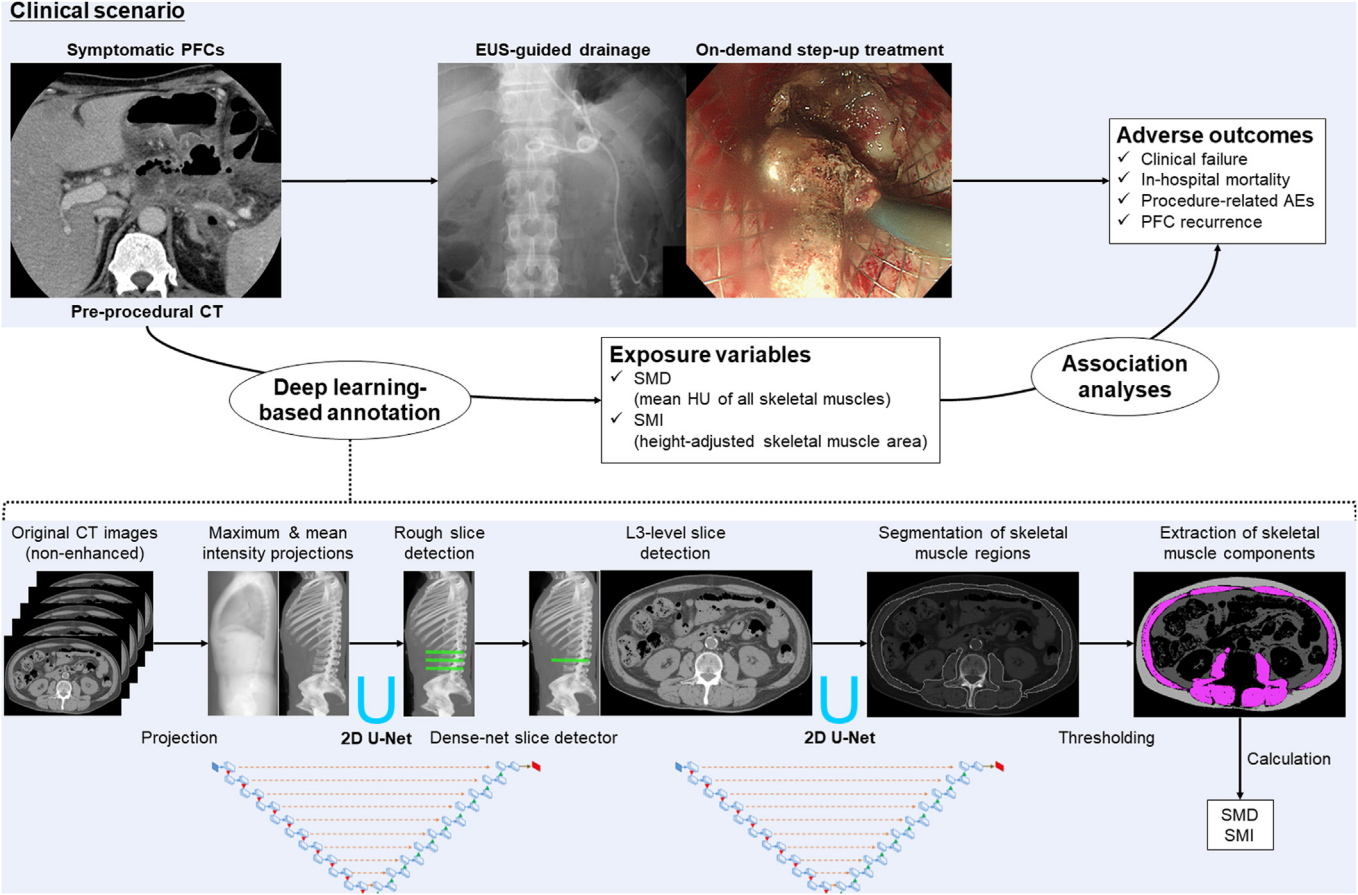
Scheme of the deep learning–based automatic assessment of skeletal muscle status using CT images. *PFC*, Pancreatic fluid collection; *AE*, adverse event; *SMD*, skeletal muscle density; *HU*, Hounsfield unit; *SMI*, skeletal muscle index; *L3*, third lumbar vertebra.

Within the WONDERFUL consortium, we have collected data on imaging studies before and after EUS-guided PFC drainage in DICOM (Digital Imaging and Communications in Medicine) format.^30^ In the current study, the axial-plane nonenhanced CT images immediately before the procedures were subjected to the analysis. An in-house deep learning pipeline based on 2 U-Nets was used for the automatic annotation of the skeletal muscles (Fig. 2). The U-Net is a convolutional neural network–based pipeline primarily used for biomedical image segmentation tasks.^31^ The architecture characterized by a U-shaped structure consists of a contracting path (encoder) for context capture and an expansive path (decoder) for precise localization with skip connections. As previously described,^29^ our pipeline enables the automatic identification of a CT image at the level of the third lumber vertebra on which the bilateral transverse processes were most clearly delineated (via the first U-Net) and the subsequent automatic annotation of all skeletal muscles included in the image (via the second U-Net). The muscle areas included the psoas; erector spinae; quadratus lumborum; transversus, abdominis, external, and internal obliques; and rectus abdominis. The study radiologist (W.G.), blinded to other clinical data, visually inspected the appropriateness of the segmentation for all cases. Subsequently, the skeletal muscles were identified based on Hounsfield unit thresholds of –29 to 150 (excluding the intramuscular marbling areas). SMD was defined as the mean Hounsfield unit value of all skeletal muscles at the index image. The low density of skeletal muscles may reflect increased intramuscular fat contents.^32^ Skeletal muscle index (SMI; square centimeters/square meters) was calculated as the summed area of the skeletal muscles divided (normalized) by the squared body height. In a secondary analysis, the SMI values were dichotomized at the sex-specific cutoff point proposed by the guidelines of Japan Society of Hepatology for sarcopenia^33^ (ie, <42 cm^2^/m^2^ for male subjects and < 38 cm^2^/m^2^ for female subjects).

### EUS-guided and adjunctive treatment of PFCs

The procedures of EUS-guided and adjunctive treatment in the WONDERFUL cohort are described in the Appendix 1. In brief, a linear array echoendoscope was inserted with the patient under conscious sedation, and EUS-guided transmural placement of a plastic or metal stent was performed. For patients who were amenable to the initial drainage, the step-up treatment was undertaken mainly via endoscopic procedures; that is, direct endoscopic necrosectomy, additional EUS-guided drainage (referred to as the multi-gateway approach^34^), and/or additional drainage through the initial puncture tract.^35^ Percutaneous drainage was considered for endoscopically nonaccessible lesions. When the symptoms were not managed through those treatment modalities, the patients were referred for salvage surgical interventions. A LAMS with electrocautery-enhanced delivery (Hot AXIOS; Boston Scientific Japan, Tokyo, Japan) was approved in October 2018 in Japan and has been used as a treatment modality for PFCs in clinical practice.

### Definitions of outcome variables and covariates

Clinical success was defined as a reduction in the PFC size to ≤2 cm or removal of all transmural and percutaneous stents/catheters with relief of symptoms associated with PFCs. Time to clinical success was defined as the time from the initial EUS-guided drainage to clinical success, the last follow-up, or death, whichever came first. For cases with clinical success, PFC recurrence was defined as the occurrence of a new PFC or exacerbation of the treated PFC on cross-sectional imaging studies. Time to PFC recurrence was defined as the time from clinical success to PFC recurrence, the last follow-up, or death, whichever came first.

Endoscopic procedure–related adverse events (eg, bleeding, peritonitis, pancreatitis) were defined and graded according to the American Society for Gastrointestinal Endoscopy lexicon guidelines.^36^ The criteria were extrapolated to adverse events associated with percutaneous interventions. A PFC-related death was defined as a death due to PFC-related symptoms (eg, sepsis), adverse events associated with PFC treatment, or underlying pancreatitis.

### Statistical analysis

Our primary hypothesis testing was an assessment of a linear trend toward the risk of adverse outcomes of EUS-guided PFC treatment by skeletal muscle status (SMI or SMD) in the multivariable logistic regression model. Stratum-specific risk estimates represented secondary analyses. To calculate odds ratios (ORs) and 95% confidence intervals (CIs) for an adverse outcome according to SMI or SMD, the logistic regression model was used. To adjust for potential confounding factors, the multivariable logistic regression model initially included the set of covariates described in the corresponding tables. A backward elimination with the threshold *P* of .05 was conducted to select variables for the final models. For cases with missing data on body mass index (2.0%), we assigned the median value and subsequently confirmed that excluding cases with missing data did not alter our findings substantially (data not shown). To examine a potential nonlinear relationship between skeletal muscle status and adverse outcomes, the restricted cubic spline function with 4 knots (5th, 35th, 65th, and 95th percentiles) and the reference value of the median value with adjustment for the aforementioned set of covariates were used.^37^

Tests for overall and nonlinear relationships between skeletal muscle status and the outcomes were performed by using the χ^2^ test. In analyses stratified according to PFC type, a statistical interaction was assessed by using the Wald test on the cross-product of skeletal muscle status (continuous) and stent types (WON vs non-necrotic PFCs [pseudocysts and postoperative PFCs]) in the logistic regression model. Stratum-specific ORs were calculated based on a single regression model with a reparameterization of the interaction term.^38^ Cumulative incidence functions of clinical success and PFC recurrence were estimated via the competing risk framework, in which competing risk events included salvage surgery and death for clinical success and death for PFC recurrence.^39^^,^^40^ Patients who were lost to follow-up were dealt with as censored cases at the time of the last follow-up. Cumulative incidence functions were compared between groups via Gray’s test using SAS version 9.4 (SAS Institute, Cary, NC, USA).

To compare characteristics between the subgroups, the χ^2^ test or Fisher exact test, as appropriate, was used for categorical variables, and the analysis of variance or the Kruskal-Wallis test, as appropriate, was used for continuous variables.

All statistical analyses were performed by using the Stata version 18 (StataCorp LLC, College Station, Tex, USA) unless otherwise noted. All *P* values were 2-sided. Given multiple comparisons, the stringent 2-sided α level of .005 was used for statistical significance.^41^

## Results

**Supplementary Figure 1:**
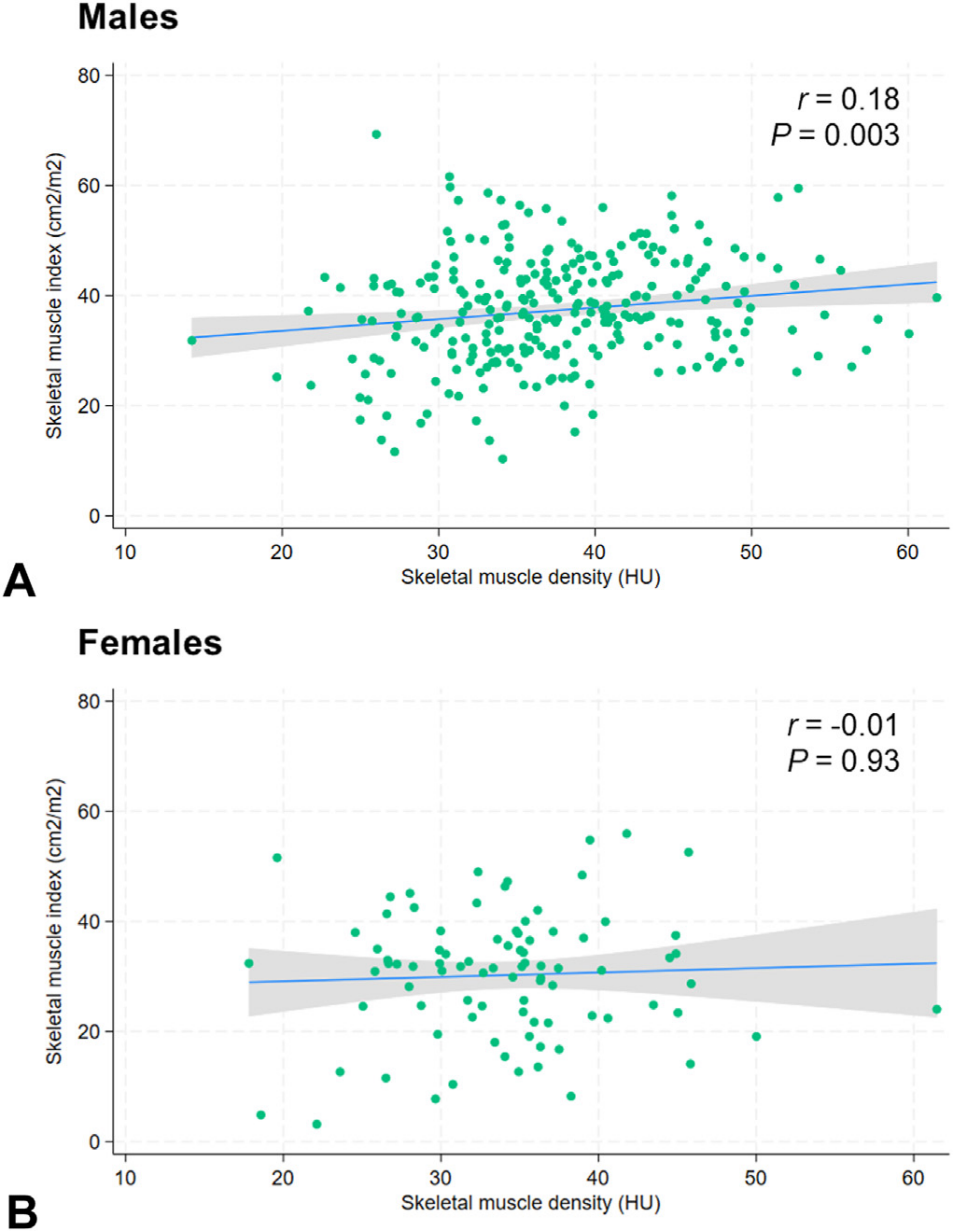
Correlation plots of skeletal muscle density and skeletal muscle index according to sex. The correlation coefficient (*r*) and *P* value were calculated by using the Spearman correlation test. The gray area indicates the 95% confidence interval of the regression line (*solid line*). **A,** Male subjects. **B,** Female subjects. *HU*, Hounsfield unit.

We analyzed 372 patients receiving EUS-guided treatment of PFCs (218, 107, and 47 patients with WON, pseudocysts, and postoperative PFCs, respectively) (Fig. 1). Table 1 and Supplementary Table 1 (available online at www.igiejournal.org) summarize the clinical characteristics of the patients according to the levels of SMD and SMI, respectively. Patients with lower levels of SMD were more likely to be aged and have WON, a high white blood cell count, and low albumin levels. The levels of SMI were positively associated with body mass index. In the total study population, low SMI levels based on the cutoff point in the definition of sarcopenia were observed in 275 (74.7%) patients. The correlation of SMD and SMI was weak, with Spearman correlation coefficients of .18 and –.01 for male and female subjects, respectively (Supplementary Fig. 1, available online at www.igiejournal.org). Clinical failure and in-hospital mortality were observed in 59 (15.9%) and 26 (7.0%) patients, respectively.

**Table 1:** Clinical characteristics of and treatment modalities for patients receiving EUS-guided treatment of PFCs, overall or according to skeletal muscle density

Characteristic∗	All cases (N = 372)	Skeletal muscle density†				*P* value
		Q4 (highest) (n = 93)	Q3 (n = 93)	Q2 (n = 93)	Q1 (lowest) (n = 93)	
Sex						–
Male	284 (76.3)	71 (76.3)	71 (76.3)	71 (76.3)	71 (76.3)	
Female	88 (23.7)	22 (23.7)	22 (23.7)	22 (23.7)	22 (23.7)	
Age, y	60.5 ± 13.7	52.0 ± 12.4	59.4 ± 12.4	63.9 ± 11.8	66.8 ± 13.7	<.001
Year of admission						.22
2010-2013	92 (24.7)	19 (20.4)	17 (18.3)	24 (25.8)	32 (34.4)	
2014-2017	121 (32.5)	31 (33.3)	34 (36.6)	28 (30.1)	28 (30.1)	
2018-2020	159 (42.8)	43 (46.3)	42 (45.1)	41 (44.1)	33 (35.5)	
Body mass index						.18
<25 kg/m^2^	315 (85.6)	81 (88.0)	82 (88.2)	77 (84.6)	75 (81.5)	
25-29.9 kg/m^2^	43 (11.7)	10 (10.9)	11 (11.8)	11 (12.1)	11 (12.0)	
≥30 kg/m^2^	10 (2.7)	1 (1.1)	0	3 (3.3)	6 (6.5)	
Preprocedural WBC, 10^9^/L	8.9 (6.2-13.3)	7.8 (5.8-11.0)	8.1 (5.9-12.7)	10.4 (6.8-14.3)	10.0 (6.7-13.9)	.004
Preprocedural albumin, mg/dL	2.8 (2.3-3.3)	3.3 (2.8-3.9)	2.8 (2.3-3.2)	2.7 (2.2-3.1)	2.4 (2.0-2.9)	<.001
Type of PFC						<.001
Walled-off necrosis	218 (58.6)	38 (40.9)	55 (59.1)	56 (60.2)	69 (74.2)	
Pseudocyst	107 (28.8)	43 (46.2)	28 (30.1)	20 (21.5)	16 (17.2)	
Postoperative PFC	47 (12.6)	12 (12.9)	10 (10.8)	17 (18.3)	8 (8.6)	
Size of PFC, cm	10.0 (7.0-14.5)	8.1 (6.0-12.0)	9.4 (7.0-14.6)	10.9 (7.8-14.4)	11.0 (7.0-15.0)	.006
Indication of treatment						.007
Infection	239 (64.3)	42 (45.1)	63 (67.7)	65 (69.8)	69 (74.2)	
Abdominal pain	66 (17.7)	24 (25.8)	14 (15.1)	13 (14.0)	15 (16.1)	
Expanding collection	54 (14.5)	22 (23.7)	12 (12.9)	13 (14.0)	7 (7.5)	
Others	13 (3.5)	5 (5.4)	4 (4.3)	2 (2.2)	2 (2.2)	
Route of EUS-guided drainage						.64
Transgastric	344 (92.5)	88 (94.6)	87 (93.5)	87 (93.5)	82 (88.1)	
Transduodenal	25 (6.7)	5 (5.4)	5 (5.4)	5 (5.4)	10 (10.8)	
Transesophageal	3 (.8)	0	1 (1.1)	1 (1.1)	1 (1.1)	
Stent type						.42
Plastic stent	258 (69.3)	71 (76.4)	60 (64.5)	64 (68.8)	63 (67.8)	
Lumen-apposing metal stent	71 (19.1)	15 (16.1)	22 (23.7)	19 (20.4)	15 (16.1)	
Other metal stent	43 (11.6)	7 (7.5)	11 (11.8)	10 (10.8)	15 (16.1)	
Multi-gateway approach						.88
Absent	325 (87.4)	82 (88.2)	82 (88.2)	79 (84.9)	82 (88.2)	
Present	47 (12.6)	11 (11.8)	11 (11.8)	14 (15.1)	11 (11.8)	
Percutaneous drainage						.12
Absent	343 (92.2)	91 (97.8)	85 (91.4)	83 (89.2)	84 (90.3)	
Present	29 (7.8)	2 (2.2)	8 (8.6)	10 (10.8)	9 (9.7)	
Endoscopic necrosectomy						.18
Absent	265 (71.2)	73 (78.5)	64 (68.8)	68 (73.1)	60 (64.5)	
Present	107 (28.8)	20 (21.5)	29 (31.2)	25 (26.9)	33 (35.5)	

Values are n (%), mean ± standard deviation, or median (interquartile range).

*PFCs*, Pancreatic fluid collections; *Q*, quartile; *WBC*, white blood cell.

∗ Percentage indicates the proportion of cases with a specific characteristic in all cases or each stratum of skeletal muscle density.

† Levels of skeletal muscle density were categorized into sex-specific quartiles: Q1 (10.3-30.3 Hounsfield unit [HU]), Q2 (30.4-36.8 HU), Q3 (36.9-43.7 HU), and Q4 (43.8-69.3 HU) for male subjects; and Q1 (3.2-23.1 HU), Q2 (23.2-31.6 HU), Q3 (31.7-37.2 HU), and Q4 (37.3-55.9 HU) for female subjects.

**Figure 3:**
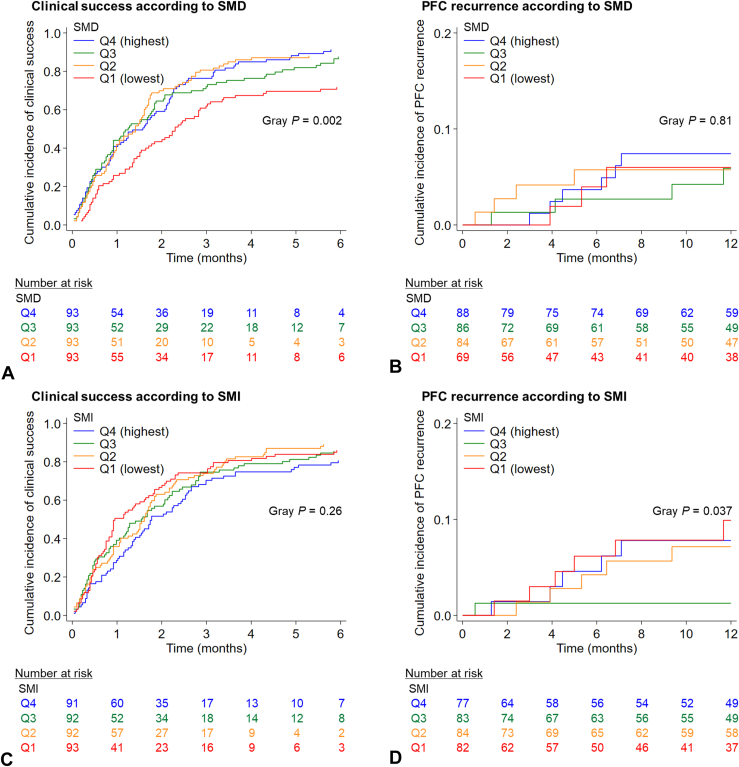
Cumulative incidence curves of clinical success and recurrence of pancreatic fluid collection (PFC) among patients receiving EUS-guided treatment of PFCs in relation to skeletal muscle status. **A,** Clinical success according to skeletal muscle density (SMD). **B,** PFC recurrence according to SMD. **C,** Clinical success according to skeletal muscle index (SMI). **D,** PFC recurrence according to SMI. *Q*, Quartile.

**Supplementary Figure 2:**
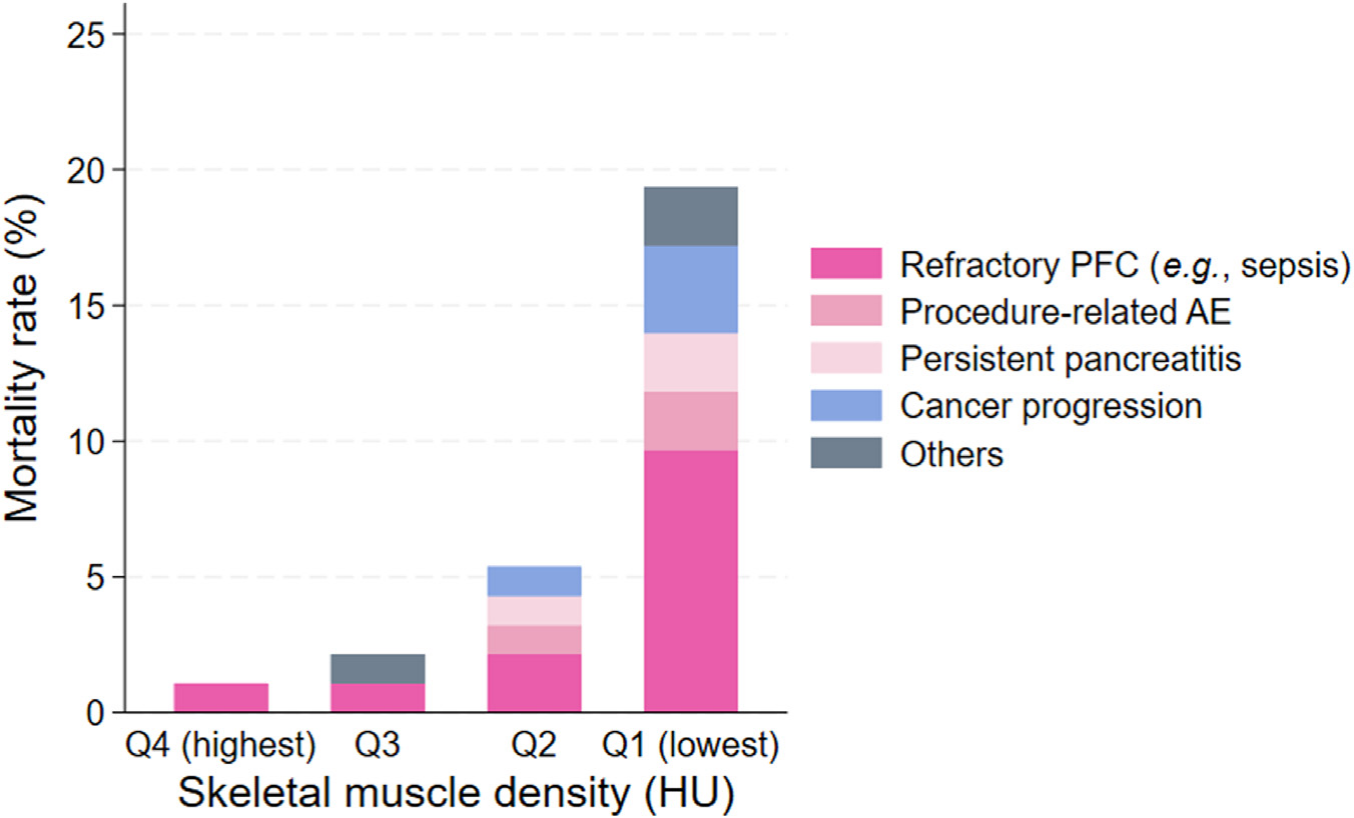
Mortality rates and causes of death according to the levels of skeletal muscle density. *PFC*, Pancreatic fluid collection; *Q*, quartile; *AE*, adverse event; *HU*, Hounsfield unit.

**Supplementary Figure 3:**
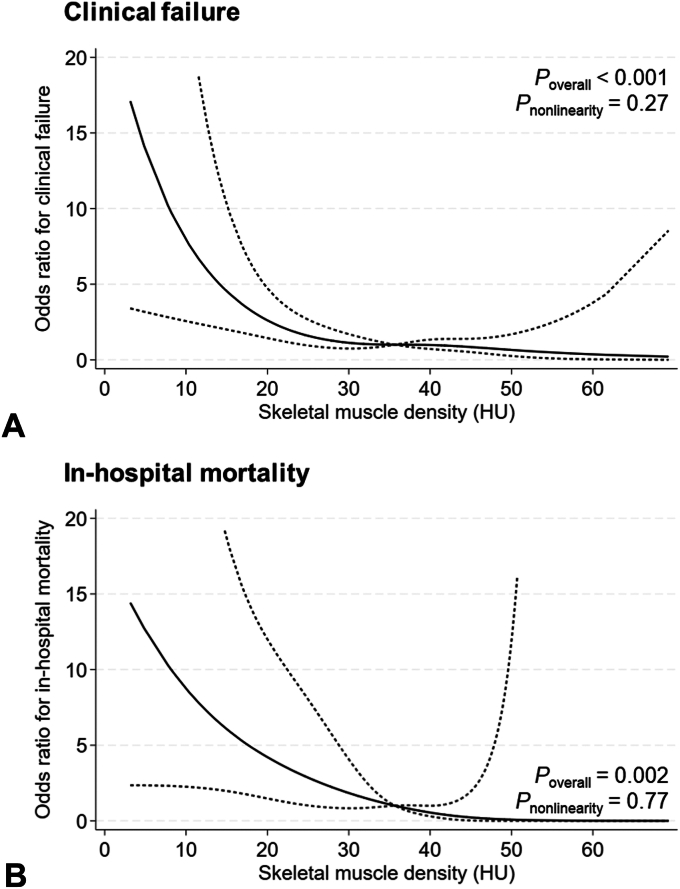
Restricted cubic spline curves for the associations of skeletal muscle density levels with adverse outcomes among patients receiving EUS-guided treatment of pancreatic fluid collections. **A,** Clinical failure. **B,** In-hospital mortality. The odds ratios were adjusted for the same set of covariates as Table 2. The *dotted lines* in each panel indicate a 95% confidence interval. *HU*, Hounsfield unit.

In our primary hypothesis testing, lower levels of SMD were associated with higher risks of clinical failure and in-hospital mortality (*P*_trend_ < .001) (Table 2; Supplementary Table 2, available online at www.igiejournal.org). The adjusted OR for clinical failure comparing the extreme quartiles of SMD was 3.64 (95% CI, 1.52-8.72). Supplementary Figure 2 (available online at www.igiejournal.org) summarizes the mortality rates and causes of death according to SMD levels. Similarly, lower levels of SMD were associated with longer time to clinical success (Fig. 3A). Compared with patients with the top 2 quartiles of SMD, patients with the lowest quartile had an adjusted OR for in-hospital mortality of 12.4 (95% CI, 3.43-44.8). A sensitivity analysis of PFC-related mortality as an outcome variable yielded similar results with the corresponding adjusted OR of 9.47 (95% CI, 2.62-34.3; *P*_trend_ < .001). When a restricted cubic spline curve for SMD was fitted in relation to clinical failure or in-hospital mortality, a risk increase was observed only within the range of very low SMD levels despite no statistically significant evidence on the nonlinear dose-response relationship (*P*_nonlinearity_ > .26) (Supplementary Fig. 3, available online at www.igiejournal.org). SMD levels were not associated with the risk of procedure-related adverse events or PFC recurrence (*P* > .17) (Fig. 3B, Table 2). In contrast, SMI levels were not associated with adverse outcomes (*P*_trend_ > .39) (Fig. 3C and D; Supplementary Table 3, available online at www.igiejournal.org). In addition, the dichotomized SMI levels based on the definition of sarcopenia were not associated with adverse outcomes (*P* > .09) (Supplementary Table 4, available online at www.igiejournal.org).

**Table 2:** Logistic regression analyses of the associations of skeletal muscle density with adverse outcomes of patients receiving EUS-guided treatment of PFCs

	Skeletal muscle density				*P*_trend_∗
	Q4 (highest) (n = 93)	Q3 (n = 93)	Q2 (n = 93)	Q1 (lowest) (n = 93)	
Clinical failure					
No. of events (%)	8 (8.6)	13 (14.0)	11 (11.8)	27 (29.0)	
Univariable OR (95% CI)	1 (referent)	1.73 (.68-4.39)	1.43 (.55-3.72)	4.35 (1.85-10.2)	<.001
Multivariable OR (95% CI)†	1 (referent)	1.44 (.55-3.77)	1.21 (.45-3.21)	3.64 (1.52-8.72)	<.001
In-hospital mortality					
No. of events (%)	1 (1.1)	2 (2.2)	5 (5.4)	18 (19.4)	
Univariable OR (95% CI)	1 (referent)		3.47 (.81-14.8)	14.6 (4.19-51.2)	<.001
Multivariable OR (95% CI)†	1 (referent)		2.75 (.63-12.1)	12.4 (3.43-44.8)	<.001
All procedure-related AEs					
No. of events (%)	9 (9.7)	15 (16.1)	22 (23.7)	13 (14.0)	
Univariable OR (95% CI)	1 (referent)	1.79 (.74-4.34)	2.89 (1.25-6.68)	1.52 (.61-3.74)	.084
Multivariable OR (95% CI)†	1 (referent)	1.40 (.56-3.49)	2.25 (.95-5.38)	.97 (.38-2.47)	.57
Bleeding					
No. of events (%)	3 (3.2)	9 (9.7)	10 (10.8)	8 (8.6)	
Univariable OR (95% CI)	1 (referent)	3.21 (.84-12.3)	3.61 (.96-13.6)	2.82 (.72-11.0)	.071
Multivariable OR (95% CI)†	1 (referent)	2.58 (.66-10.1)	3.01 (.79-11.5)	2.18 (.55-8.68)	.18
PFC recurrence‡					
No. of events (%)	10 (11.4)	7 (8.1)	6 (7.1)	4 (5.8)	
Univariable OR (95% CI)	1 (referent)	.69 (.25-1.91)	.60 (.21-1.73)	.48 (.14-1.60)	.069
Multivariable OR (95% CI)†	1 (referent)	1.13 (.38-3.38)	1.14 (.36-3.65)	1.10 (.28-4.39)	.78

*PFCs*, Pancreatic fluid collections; *Q*, quartile; *OR*, odds ratio; *CI*, confidence interval; *AEs*, adverse events.

∗ *P*_trend_ was calculated by entering skeletal muscle density (continuous) in the model.

† The multivariable logistic regression model initially included age (continuous), year of admission (continuous), body mass index (continuous), preprocedural white blood cell count (continuous), the type of PFC (walled-off necrosis vs pseudocyst vs postoperative PFC), the size of the PFC (continuous), an indication of treatment (infection vs abdominal pain vs expanding PFC vs others), the route of EUS-guided drainage (transgastric vs others), and the type of stent (plastic vs metal). The pancreatic duct status (no disruption vs partial/complete disruption) was further included in the multivariable model for PFC recurrence. Backward elimination with the threshold *P* of .10 was conducted to select variables for the final models. The variables that remained in the final models are described in Supplementary Table 2.

‡ Cases with clinical success were analyzed (n = 327).

Given the potentially heterogeneous profiles of risk factors per the etiology of PFCs,^30^ we examined the associations of SMD with clinical failure and in-hospital mortality in the strata of the PFC types (WON vs non-necrotic PFCs [pseudocysts and postoperative PFCs]) (Table 3). No effect modification by the PFC types (*P*_interaction_ > .16) was observed, but the limited number of events in the stratum of non-necrotic PFCs precluded robust statistical assessments.

**Table 3:** Logistic regression analyses of the associations of skeletal muscle density with adverse outcomes of patients receiving EUS-guided treatment of PFCs, stratified according to PFC types

	Skeletal muscle density				*P*_trend_∗	*P*_interaction_†
	Q4 (highest)	Q3	Q2	Q1 (lowest)		
Clinical failure						
WON						
No. of cases (n = 218)	38	55	56	69		
No. of events (%)	5 (13.2)	9 (16.4)	10 (17.9)	23 (33.3)		
Univariable OR (95% CI)	1 (referent)	1.26 (.43-3.67)	1.40 (.49-3.99)	3.21 (1.25-8.23)	.001	.71
Multivariable OR (95% CI)‡	1 (referent)	1.42 (.47-4.29)	1.64 (.56-4.82)	3.58 (1.36-9.42)	.002	.64
Non-necrotic PFCs						
No. of cases (n = 154)	55	38	37	24		
No. of events (%)	3 (5.5)	4 (10.5)	1 (2.7%)	4 (16.7)		
Univariable OR (95% CI)	1 (referent)	1.14 (.31-4.18)	.27 (.03-2.28)	1.94 (.52-7.32)	.18	
Multivariable OR (95% CI)‡	1 (referent)	1.40 (.37-5.30)	.27 (.03-2.35)	2.07 (.53-8.05)	.24	
In-hospital mortality						
WON						
No. of cases (n = 218)	38	55	56	69		
No. of events (%)	1 (2.6)	2 (3.6)	3 (5.4)	14 (20.3)		
Univariable OR (95% CI)	1 (referent)		1.46 (.31-6.75)	6.55 (2.06-20.9)	.001	.15
Multivariable OR (95% CI)‡	1 (referent)		1.32 (.28-6.29)	6.42 (1.92-21.4)	.001	.17
Non-necrotic PFCs						
No. of cases (n = 154)	55	38	37	24		
No. of events (%)	0	0	2 (5.4%)	4 (16.7%)		
Univariable OR (95% CI)	1 (referent)		NA	NA	.002	
Multivariable OR (95% CI)‡	1 (referent)		NA	NA	.003	

*PFCs*, Pancreatic fluid collections; *Q*, quartile; *WON*, walled-off necrosis; *OR*, odds ratio; *CI*, confidence interval; *NA*, not available.

∗ *P*_trend_ was calculated by entering skeletal muscle density (continuous) in the model.

† *P*_interaction_ was calculated by evaluating the Wald test for a cross-product term of skeletal muscle density (continuous) and PFC type (WON vs non-necrotic PFCs) in the multivariable logistic regression model.

‡ The multivariable logistic regression model initially included age (continuous), year of admission (continuous), body mass index (continuous), the type of PFC (WON vs pseudocyst vs postoperative PFC), the size of PFC (continuous), an indication of treatment (infection vs abdominal pain vs expanding PFC vs others), the route of EUS-guided drainage (transgastric vs others), and the type of stent (plastic vs metal). The pancreatic duct status (no disruption vs partial/complete disruption) was further included in the multivariable model for PFC recurrence. Backward elimination with the threshold *P* of .10 was conducted to select variables for the final models.

## Discussion

In this multi-institutional series of patients receiving EUS-guided PFC treatment, we have shown that lower levels of SMD (as a surrogate for poorer skeletal muscle quality) may be associated with higher risks of clinical treatment failure and in-hospital mortality. These findings were consistently observed in the strata of the PFC types. In contrast, the SMI levels (as a surrogate for skeletal muscle volume) were not associated with the risk of adverse outcomes in this study population. Our data support not only the potential of SMD in identifying patients at high risk of adverse outcomes but also the necessity of optimizing management strategies for patients with low levels of SMD.

The reliable annotation of skeletal muscle areas in cross-sectional abdominal images is a prerequisite for clinical research on skeletal muscle status in relation to disease outcomes. Most studies have used analytical software that requires manual outlining of muscle fat boundaries.^21^ However, this time-consuming manual segmentation has inherent bias derived from nonautomated and thus operator-dependent procedures (eg, selecting a representative image at a given body level and identifying all skeletal muscle areas while excluding non-muscle components). In contrast to the prior studies, the current study was merited by the utilization of AI-based automatic annotation of skeletal muscles. Deep learning–based image recognition has emerged as a platform of AI, which is increasingly used as a means of identifying abnormal findings from a series of medical images in an automatic unbiased fashion.^42^^,^^43^ This study supports the applicability of AI-based image data extraction to large-scale cohort studies.

Using our AI-based profiling of body composition, the current study indicates that patients with lower levels of SMD may be more likely to experience clinical failure and in-hospital mortality during the treatment period of PFCs. The adverse impact of low-level SMD on clinical outcomes can be attributed to several plausible reasons. Skeletal muscle status might represent a mutually dependent relationship with systemic inflammatory and immune status; that is, skeletal muscle dysregulation not only results from sustained inflammation and malnutrition but also hampers immune responses.^24^^,^^25^ The dysregulated muscle status could reflect decreased physiological reserve, which could increase the risk of mortality in case of severe adverse events such as sepsis and perforation of the PFC wall. However, the SMD levels seemed not to be associated with the risk of procedure-related adverse events; therefore, the elevated risk of adverse events per se may not explain the inverse association of SMD and the risk of adverse outcomes. Lower levels of SMD were associated with a higher risk of PFC-related mortality as well as that of overall mortality and a longer time to clinical success. Therefore, it is considered that patients with impaired skeletal muscle quality may more likely become intolerant to adjunctive interventions after the initial drainage owing to the worsened systemic conditions. Considering evidence suggesting an inverse relationship between hospital caseloads and the risk of adverse outcomes,^44^^,^^45^ the referral of patients with low levels of SMD to high-volume centers may be recommended. A further investigation is warranted to examine whether muscle-modulating programs (eg, early rehabilitation and specific nutritional interventions^17^^,^^46^) can result in those muscular parameters and thereby improve the clinical outcomes of patients receiving EUS-guided PFC treatment.

The null associations of SMI levels with clinical outcomes of EUS-guided PFC treatment require comments. Our data implicate the limited predictive ability of SMI in an investigation using the cutoff value for sarcopenia proposed in the guideline as well as in an investigation of a linear trend. Given the remarkable prognostic role noted in SMD, these findings might be due to chance, but there are several possible explanations. In line with the published data,^19^ the guideline-defined muscle loss corresponding to sarcopenia was observed in a majority of patients in our cohort (ie, 70%), presumably owing to prolonged fasting and sustained infection and inflammation. Therefore, the prognostic role of SMI might be largely diluted focusing on a group of patients with endoscopically treated PFCs. Mechanistically, the pathogenic pathways and related molecules may not be completely overlapped for decreased levels of skeletal muscle quality and quantity.^25^ In translational research on patients with periampullary malignancy, distinctive profiles of intramuscular gene expressions and circulating metabolites were shown for low levels of SMI and SMD defined on the basis of CT images.^47^ Further research is warranted to clarify the biological mechanism through which skeletal muscle quality, but not the quantity, can affect the clinical outcomes of this patient population to develop a new treatment strategy.

The current study has notable strengths. Our large sample size accumulated in the multicenter setting provided robust statistical assessments and potentially increased the generalizability of the findings. In addition, the detailed information on the demographic characteristics and procedures allowed us to rigorously adjust for a variety of potential confounders in the multivariable analyses. More importantly, our deep learning–based annotation of skeletal muscle areas minimized potential biases due to measurement errors in the skeletal muscle metrics.

The current study also has several limitations. First, there was a possibility of unmeasured confounding factors in the multivariable models; nonetheless, we adjusted for a variety of clinical parameters, including known risk factors for adverse outcomes at various therapeutic phases,^30^ and the adjustment did not alter the results substantially. Second, a vast majority of the study population was Japanese. Given the different body compositions between race groups, our findings on the prognostic roles of skeletal muscle metrics should be validated in independent populations with racial diversity.

In conclusion, SMD (quality) may provide valuable information for the risk stratification of patients who are scheduled to receive EUS-guided PFC treatment. Given the invasiveness of EUS-guided drainage and adjunctive treatment in the management of symptomatic PFCs, the current study highlights the importance of assessing the skeletal muscle quality of patients before the interventions. Further research is required to elucidate how patients with a high-risk muscular profile should be managed and whether physical and nutritional programs aimed at improving muscular status can reduce adverse outcomes.

## Disclosure

The following authors disclosed financial relationships: H. Isayama: research funding from 10.13039/100016242Boston Scientific Japan, 10.13039/501100002424Fujifilm, and Piolax Medical Devices; honoraria from Boston Scientific Japan, Century Medical, Create Medic, Fujifilm, Gadelius Medical, Hitachi Medical, Japan Lifeline, Kaneka, Kawasumi Laboratories, Olympus Medical, Piolax Medical Devices, Sumitomo Bakelite, UMIDAS, and Zeon Medical; and contributions from Boston Scientific Japan, Gadelius Medical, Japan Lifeline, and Zeon Medical. Y. Nakai: research funding from Boston Scientific Japan, Century Medical, Fujifilm, Gadelius Medical, Hitachi Medical, HOYA Pentax Medical, Kaneka, and Medico’s Hirata; and honoraria from Boston Scientific Japan, Fujifilm, Gadelius Medical, Hitachi Medical, J-MIT, Medico’s Hirata, and Olympus Medical. This work was not supported by any of those companies. All other authors disclosed no financial relationships. This work was supported by grants from the 10.13039/100018254Japanese Foundation for Research and Promotion of Endoscopy (to Drs Saito and Nakai). T. Hamada was supported by a KAKENHI grant from the Japan Society for the Promotion of Science (JP22H02841) and by grants from 10.13039/100007449Takeda Science Foundation and 10.13039/501100002336Daiichi Sankyo Company. The funders had no role in study design, data collection and analysis, the decision to publish, or the preparation of the manuscript.

## Figures and Tables

**Supplementary Table 1:** Clinical characteristics of and treatment modalities for patients receiving EUS-guided treatment of PFCs, overall or according to skeletal muscle index

Characteristic∗	All cases (N = 368)	Skeletal muscle index†				*P* value
		Q4 (highest) (n = 91)	Q3 (n = 92)	Q2 (n = 92)	Q1 (lowest) (n = 93)	
Sex						–
Male	281 (76.4)	70 (76.9)	70 (76.1)	70 (76.1)	71 (76.3)	
Female	87 (23.6)	21 (23.1)	22 (23.9)	22 (23.9)	22 (23.7)	
Age, y	60.5 ± 13.7	58.3 ± 13.3	62.6 ± 12.7	61.0 ± 12.4	59.9 ± 16.0	.19
Year of admission						.20
2010-2013	90 (24.5)	27 (29.7)	22 (23.9)	20 (21.7)	21 (22.6)	
2014-2017	120 (32.6)	31 (34.1)	26 (28.3)	38 (41.3)	25 (26.9)	
2018-2020	158 (42.9)	33 (36.2)	44 (47.8)	34 (37.0)	47 (50.5)	
Body mass index						<.001
<25 kg/m^2^	315 (85.6)	58 (63.7)	82 (89.1)	84 (91.3)	91 (97.8)	
25-29.9 kg/m^2^	43 (11.7)	25 (27.5)	9 (9.8)	8 (8.7)	1 (1.1)	
≥30 kg/m^2^	10 (2.7)	8 (8.8)	1 (1.1)	0	1 (1.1)	
Preprocedural WBC, 10^9^/L	8.9 (6.1-13.3)	8.3 (6.3-13.9)	7.8 (5.8-12.3)	10.0 (6.4-13.6)	8.8 (6.2-13.3)	.39
Preprocedural albumin, mg/dL	2.8 (2.3-3.3)	2.8 (2.3-3.5)	2.8 (2.4-3.4)	2.8 (2.3-3.3)	2.7 (2.2-3.2)	.48
Type of PFC						.008
Walled-off necrosis	217 (59.0)	57 (62.6)	59 (64.1)	58 (63.1)	43 (46.2)	
Pseudocyst	105 (28.5)	25 (27.5)	18 (19.6)	21 (22.8)	41 (44.1)	
Postoperative PFC	46 (12.5)	9 (9.9)	15 (16.3)	13 (14.1)	9 (9.7)	
Size of PFC, cm	10.0 (7.0-14.5)	10.2 (7.5-15.2)	10.6 (7.0-14.6)	10.0 (6.6-14.0)	8.5 (6.2-13.0)	.090
Indication of treatment						.17
Infection	236 (64.2)	59 (64.8)	55 (59.8)	61 (66.3)	61 (65.6)	
Abdominal pain	66 (17.9)	13 (14.3)	21 (22.8)	19 (20.7)	13 (14.0)	
Expanding collection	53 (14.4)	17 (18.7)	13 (14.1)	6 (6.5)	17 (18.3)	
Others	13 (3.5)	2 (2.2)	3 (3.3)	6 (6.5)	2 (2.2)	
Route of EUS-guided drainage						.57
Transgastric	341 (92.7)	84 (92.3)	83 (90.2)	87 (94.6)	87 (93.5)	
Transduodenal	24 (6.5)	7 (7.7)	7 (7.6)	4 (4.3)	6 (6.5)	
Transesophageal	3 (.8)	0	2 (2.2)	1 (1.1)	0	
Stent type						.43
Plastic stent	255 (69.3)	64 (70.3)	68 (73.9)	67 (72.8)	56 (60.2)	
Lumen-apposing metal stent	71 (19.3)	17 (18.7)	17 (18.5)	15 (16.3)	22 (23.7)	
Other metal stent	42 (11.4)	10 (11.0)	7 (7.6)	10 (10.9)	15 (16.1)	
Multi-gateway approach						.66
Absent	321 (87.2)	76 (83.5)	81 (88.0)	81 (88.0)	83 (89.2)	
Present	47 (12.8)	15 (16.5)	11 (12.0)	11 (12.0)	10 (10.8)	
Percutaneous drainage						.064
Absent	340 (92.4)	80 (87.9)	83 (90.2)	90 (97.8)	87 (93.5)	
Present	28 (7.6)	11 (12.1)	9 (9.8)	2 (2.2)	6 (6.5)	
Endoscopic necrosectomy						.061
Absent	261 (70.9)	66 (72.5)	61 (66.3)	59 (64.1)	75 (80.6)	
Present	107 (29.1)	25 (27.5)	31 (33.7)	33 (35.9)	18 (19.4)	

Values are n (%), mean ± standard deviation, or median (interquartile range).

*PFCs*, Pancreatic fluid collections; *Q*, quartile; *WBC*, white blood cell.

∗ Percentage indicates the proportion of cases with a specific characteristic in all cases or each stratum of skeletal muscle index. Total percentages may not equal 100% due to rounding.

† Levels of skeletal muscle index were categorized into sex-specific quartiles: Q1 (14.2-32.6 cm^2^/m^2^), Q2 (32.7-36.9 cm^2^/m^2^), Q3 (37.0-42.6 cm^2^/m^2^), and Q4 (42.7-61.8 cm^2^/m^2^) for male subjects; and Q1 (17.8-29.7 cm^2^/m^2^), Q2 (29.8-34.5 cm^2^/m^2^), Q3 (34.6-37.1 cm^2^/m^2^), and Q4 (37.2-61.5 cm^2^/m^2^) for female subjects.

**Supplementary Table 2:** Logistic regression analyses of the associations of skeletal muscle density with adverse outcomes of patients receiving EUS-guided treatment of PFCs (the final multivariable models)

	Multivariable OR (95% CI)∗ for an adverse outcome
Clinical failure	
Skeletal muscle density	
Q4 (highest)	1 (referent)
Q3	1.44 (.55-3.77)
Q2	1.21 (.45-3.21)
Q1 (lowest)	3.64 (1.52-8.72)
Size of PFC (per 5-cm increase)	1.73 (1.31-2.30)
Stent type	
Metal stent including LAMS	1 (referent)
Plastic stent	1.69 (.87-3.30)
In-hospital mortality	
Skeletal muscle density	
Q4-Q3 (highest)	1 (referent)
Q2	2.75 (.63-12.1)
Q1 (lowest)	12.4 (3.43-44.8)
Size of PFC (per 5-cm increase)	1.75 (1.16-2.65)
Indication of treatment	
Infection	1 (referent)
Others	.08 (.01-.58)
All procedure-related adverse events	
Skeletal muscle density	
Q4 (highest)	1 (referent)
Q3	1.40 (.56-3.49)
Q2	2.25 (.95-5.38)
Q1 (lowest)	.97 (.38-2.47)
Type of PFC	
Walled-off necrosis	1 (referent)
Pseudocyst	.17 (.06-.45)
Postoperative PFC	.28 (.10-.84)
Bleeding	
Skeletal muscle density	
Q4 (highest)	1 (referent)
Q3	2.58 (.66-10.1)
Q2	3.01 (.79-11.5)
Q1 (lowest)	2.18 (.55-8.68)
Size of PFC (per 5-cm increase)	1.67 (1.19-2.36)
PFC recurrence†	
Skeletal muscle density	
Q4 (highest)	1 (referent)
Q3	1.13 (.38-3.38)
Q2	1.14 (.36-3.65)
Q1 (lowest)	1.10 (.28-4.39)
Age (per 10-year increase)	.66 (.48-.91)
Type of PFC	
Walled-off necrosis	1 (referent)
Pseudocyst or postoperative PFC	2.57 (1.07-6.22)
Route of EUS-guided drainage	
Transgastric	1 (referent)
Others	.30 (.10-.95)

*PFCs*, Pancreatic fluid collections; *OR*, odds ratio; *CI*, confidence interval; *Q*, quartile; *LAMS*, lumen-apposing metal stent.

∗ The multivariable logistic regression model initially included age (continuous), year of admission (continuous), body mass index (continuous), the type of PFC (walled-off necrosis vs pseudocyst vs postoperative PFC), the size of PFC (continuous), an indication of treatment (infection vs abdominal pain vs expanding PFC vs others), the route of EUS-guided drainage (transgastric vs others), and the type of stent (plastic vs metal). The pancreatic duct status (no disruption vs partial/complete disruption) was further included in the multivariable model for PFC recurrence. Backward elimination with the threshold *P* of .10 was conducted to select variables for the final models.

† Cases with clinical success were analyzed (n = 327).

**Supplementary Table 3:** Logistic regression analyses of the associations of skeletal muscle index with adverse outcomes of patients receiving EUS-guided treatment of PFCs

	Skeletal muscle index				*P*_trend_∗
	Q4 (highest) (n = 91)	Q3 (n = 92)	Q2 (n = 92)	Q1 (lowest) (n = 93)	
Clinical failure					
No. of events (%)	19 (20.9)	14 (15.2)	10 (10.9)	13 (14.0)	
Univariable OR (95% CI)	1 (referent)	.68 (0.32-1.46)	.46 (.20-1.06)	.62 (.28-1.34)	.16
Multivariable OR (95% CI)†	1 (referent)	.69 (0.31-1.54)	.47 (.20-1.11)	.82 (.36-1.87)	.50
In-hospital mortality					
No. of events (%)	8 (8.8)	4 (4.3)	5 (5.4)	7 (7.5)	
Univariable OR (95% CI)	1 (referent)	.47 (.14-1.62)	.60 (.19-1.90)	.84 (.29-2.43)	.27
Multivariable OR (95% CI)†	1 (referent)	.57 (.15-2.17)	.80 (.23-2.85)	1.42 (.39-5.10)	.40
All procedure-related AEs					
No. of events (%)	10 (11.0)	16 (17.4)	19 (20.7)	13 (14.0)	
Univariable OR (95% CI)	1 (referent)	1.71 (.73-3.99)	2.11 (.92-4.83)	1.32 (.55-3.17)	.71
Multivariable OR (95% CI)†	1 (referent)	1.68 (.71-4.00)	2.15 (.92-5.01)	1.70 (.69-4.22)	.40
Bleeding					
No. of events (%)	6 (6.6)	8 (8.7)	9 (9.8)	6 (6.5)	
Univariable OR (95% CI)	1 (referent)	1.35 (.45-4.06)	1.54 (.52-4.51)	.98 (.30-3.15)	.65
Multivariable OR (95% CI)†	1 (referent)	1.37 (.44-4.24)	1.82 (.59-5.57)	1.47 (.43-4.95)	.74
PFC recurrence‡					
No. of events (%)	7 (9.1)	1 (1.2)	10 (11.9)	9 (11.0)	
Univariable OR (95% CI)	1 (referent)	.12 (.01-1.02)	1.35 (.49-3.75)	1.23 (.44-3.49)	.39
Multivariable OR (95% CI)†	1 (referent)	.10 (.01-1.02)	2.90 (.90-9.29)	1.19 (.37-3.82)	.41

*PFCs*, Pancreatic fluid collections; *Q*, quartile; *OR*, odds ratio; *CI*, confidence interval; *AEs*, adverse events.

∗ *P*_trend_ was calculated by entering skeletal muscle index (continuous) in the model.

† The multivariable logistic regression model initially included age (continuous), year of admission (continuous), body mass index (continuous), the type of PFC (walled-off necrosis vs pseudocyst vs postoperative PFC), the size of PFC (continuous), an indication of treatment (infection vs abdominal pain vs expanding PFC vs others), the route of EUS-guided drainage (transgastric vs others), and the type of stent (plastic vs metal). The pancreatic duct status (no disruption vs partial/complete disruption) was further included in the multivariable model for PFC recurrence. Backward elimination with the threshold *P* of .10 was conducted to select variables for the final models.

‡ Cases with clinical success were analyzed (n = 326).

**Supplementary Table 4:** Logistic regression analyses of the associations of dichotomized skeletal muscle index levels with adverse outcomes of patients receiving EUS-guided treatment of PFCs

	Skeletal muscle index∗		*P* value
	High (n = 93)	Low (n = 275)	
Clinical failure			
No. of events (%)	20 (21.5)	36 (13.1)	
Univariable OR (95% CI)	1 (referent)	.55 (.30-1.01)	.053
Multivariable OR (95% CI)†	1 (referent)	.59 (.31-1.11)	.099
In-hospital mortality			
No. of events (%)	8 (8.6)	16 (5.8)	
Univariable OR (95% CI)	1 (referent)	.66 (.27-1.59)	.35
Multivariable OR (95% CI)†	1 (referent)	.66 (.26-1.70)	.39
All procedure-related AEs			
No. of events (%)	11 (11.8%)	47 (17.1%)	
Univariable OR (95% CI)	1 (referent)	1.54 (.76-3.10)	.23
Multivariable OR (95% CI)†	1 (referent)	1.63 (.80-3.35)	.18
Bleeding			
No. of events (%)	6 (6.5%)	23 (8.4%)	
Univariable OR (95% CI)	1 (referent)	1.32 (.52-3.36)	.56
Multivariable OR (95% CI)†	1 (referent)	1.57 (.60-4.11)	.36
PFC recurrence‡			
No. of events (%)	7 (8.9%)	20 (8.1%)	
Univariable OR (95% CI)	1 (referent)	.91 (.37-2.23)	.83
Multivariable OR (95% CI)†	1 (referent)	1.02 (.39-2.69)	.97

*PFCs*, Pancreatic fluid collections; *OR*, odds ratio; *CI*, confidence interval; *AEs*, adverse events.

∗ Skeletal muscle index levels were dichotomized at the sex-specific cutoff point proposed by the guidelines of the Japan Society of Hepatology for sarcopenia^9^: that is, <42 cm^2^/m^2^ for male subjects and <38 cm^2^/m^2^ for female subjects.

† The multivariable logistic regression model initially included age (continuous), year of admission (continuous), body mass index (continuous), the type of PFC (walled-off necrosis vs pseudocyst vs postoperative PFC), the size of PFC (continuous), an indication of treatment (infection vs abdominal pain vs expanding PFC vs others), the route of EUS-guided drainage (transgastric vs others), and the type of stent (plastic vs metal). The pancreatic duct status (no disruption vs partial/complete disruption) was further included in the multivariable model for PFC recurrence. Backward elimination with the threshold *P* of .10 was conducted to select variables for the final models.

‡ Cases with clinical success were analyzed (n = 326).

## Appendix 1

### Participating centers

The participating centers of the WONDERFUL (WON and Peripancreatic Fluid Collection) consortium study group were as follows: Gifu Municipal Hospital, Gifu; Gifu Prefectural General Medical Center, Gifu; Gifu University Hospital, Gifu; Hyogo Medical University Hospital, Hyogo; Juntendo University Hospital, Tokyo; Kindai University Hospital, Osaka; Kobe University Hospital, Hyogo; Saitama Medical Center, Saitama; The University of Tokyo Hospital, Tokyo; and Toyama University Hospital, Toyama (all in Japan).

### EUS-guided and adjunctive treatment of pancreatic fluid collections

Despite variations between the participating centers, endoscopic procedures during the study period were standardized to some extent. Contrast-enhanced CT imaging was conducted to evaluate the overall architecture of a pancreatic fluid collection (PFC) as well as the levels of PFC encapsulation and the possibility of a pseudoaneurysm located in the puncture route unless there were contraindications. A linear-array echoendoscope was inserted with the patient under moderate sedation, and transmural placement of a plastic or metal stent was performed with endosonographic and fluoroscopic guidance. We used double-pigtail plastic stent(s) until an LAMS with electrocautery-enhanced delivery (Hot AXIOS; Boston Scientific Japan, Tokyo, Japan) was approved in October 2018 in Japan; we used the LAMS as a first-choice option thereafter (particularly for cases with walled-off necrosis or large-size pseudocysts). Before the approval of the LAMS, a biflanged metal stent (Niti-S Nagi; Taewoong Medical, Gyeonggi-do, South Korea)^1^ was used as a clinical trial at some centers. The types of stents were selected at the endoscopist’s discretion with the availability at the center.

For patients who were amenable to the initial drainage alone, the step-up treatment was undertaken mainly via endoscopic procedures; that is, endoscopic necrosectomy, additional EUS-guided drainage (referred to as the multi-gateway approach),^2^ and/or additional drainage through the initial puncture tract.^3^ Percutaneous drainage was considered as a step-up approach for PFC lesions apart from the initially targeted PFC and the gastroduodenal lumen. When the symptoms were not managed through those treatment modalities, the patients were referred for salvage surgical interventions (eg, debridement, drainage, cystogastrostomy).
